# Plasma YKL-40 is associated with prognosis in patients with metastatic pancreatic cancer receiving immune checkpoint inhibitors in combination with radiotherapy

**DOI:** 10.3389/fimmu.2023.1228907

**Published:** 2023-09-07

**Authors:** Astrid Z. Johansen, Sif I. Novitski, Jessica X. Hjaltelin, Susann Theile, Mogens K. Boisen, Søren Brunak, Daniel H. Madsen, Dorte L. Nielsen, Inna M. Chen

**Affiliations:** ^1^ National Center for Cancer Immune Therapy, Department of Oncology, Copenhagen University Hospital – Herlev and Gentofte, Herlev, Denmark; ^2^ Novo Nordisk Foundation Center for Protein Research, Faculty of Health and Medical Sciences, University of Copenhagen, Copenhagen, Denmark; ^3^ Department of Oncology, Copenhagen University Hospital – Herlev and Gentofte, Herlev, Denmark; ^4^ Department of Immunology and Microbiology, Faculty of Health and Medical Sciences, University of Copenhagen, Copenhagen, Denmark; ^5^ Department of Clinical Medicine, Faculty of Health and Medical Sciences, University of Copenhagen, Copenhagen, Denmark

**Keywords:** biomarker, immune checkpoint inhibition, CHI3L1, pancreatic cancer, YKL-40

## Abstract

**Background:**

YKL-40, also known as chitinase-3-like protein 1 (CHI3L1), is a secreted glycoprotein produced by various cell types including stromal, immune, and cancer cells. It contributes to cancer progression through tumor-promoting inflammation and has been shown to inhibit the cytotoxicity of T and NK lymphocytes. *In vivo* studies have demonstrated synergistic anti-cancer effects of blocking YKL-40 in combination with immune checkpoint inhibitors (ICIs). Biomarkers for the prediction of the response to ICIs are highly needed. We investigated the association between plasma YKL-40 and clinical benefit and survival in patients with metastatic pancreatic cancer (mPC) receiving ICIs and stereotactic body radiotherapy (SBRT).

**Methods:**

Blood samples were collected from 84 patients with mPC who participated in the randomized phase II CheckPAC study, in which patients received nivolumab with or without ipilimumab combined with a single fraction of SBRT. Plasma YKL-40 was measured using a commercial ELISA kit.

**Results:**

Elevated baseline plasma YKL-40 was an independent predictor of shorter overall survival (OS) (HR 2.19, 95% CI 1.21–3.95). A ≥ 40% decrease in plasma YKL-40 during treatment was associated with longer progression-free survival (*p* = 0.009) and OS (*p* = 0.0028). There was no correlation between plasma YKL-40 and the tumor burden marker CA19-9 at baseline or during treatment.

**Conclusion:**

This study contributes new knowledge regarding YKL-40 as a predictor of clinical benefit from ICIs and radiotherapy. These exploratory results warrant further investigation of YKL-40 as a biomarker for patients treated with immunotherapies.

**Clinical trial registration:**

Clinicaltrials.gov, identifier NCT02866383.

## 1 Introduction

YKL-40 (also known as chitinase-3-like protein 1 [CHI3L1]) is a secreted glycoprotein that binds chitin, heparin, and collagen but lacks enzymatic activity (1). The protein is secreted by various cell types including immune (mainly macrophages and neutrophils), cancer, and stromal cells (2, 3). The protein has been linked to cancer-promoting characteristics such as stimulation of cell growth, protection against apoptosis, increased angiogenesis, and extracellular matrix remodeling (1, 2, 4, 5). YKL-40 is also suggested to contribute to an immunosuppressive tumor microenvironment by inhibiting the function of cytotoxic T and natural killer (NK) lymphocytes (6–8), stimulating programmed death-ligand 1 (PD-L1) expression (9), and regulating macrophage polarization toward an anti-inflammatory phenotype (10). Recently, *in vivo* studies using antibody-based blockade of YKL-40 or blockade using chitooligosaccharides have shown synergistic effects with immunotherapy (3, 8, 9, 11). Plasma YKL-40 is investigated as a biomarker in different diseases characterized by inflammation, including cancer, autoimmune, fibrotic, and neurodegenerative diseases (12–16). Additionally, plasma YKL-40 levels in healthy subjects increase with age (17). Higher plasma levels of YKL-40 are found in patients with cancer compared to those of healthy individuals (12). Elevated plasma YKL-40 levels are associated with shorter overall survival (OS) in patients with different cancers (13) including pancreatic cancer (PC) (18–21). Plasma YKL-40 has therefore been suggested as a prognostic cancer biomarker.

PC is an aggressive cancer type with one of the lowest 5-year survival rates - less than 10% for combined disease stages (22). PC is characterized by high tumor heterogeneity, and a collagen-dense, hypoxic, and highly immunosuppressive tumor microenvironment (23). Standard treatment options include surgery and chemotherapy, but 80% of the patients are not eligible for surgery due to advanced or metastatic disease at the time of diagnosis (24). The increasing incidence of PC related to changes in risk factors such as diabetes, obesity, tobacco usage, and alcohol consumption combined with few advances in treatment strategies have led to an urgent need for new treatment strategies (25). Currently, ICIs are not standard treatment options for patients with metastatic PC (mPC) and are only recommended for patients with tumors displaying microsatellite instability or mismatch repair deficiency (26, 27). While several biomarkers, including interleukin (IL)-6, are still under investigation for clinical applications, carbohydrate antigen 19-9 (CA19-9) remains currently the only routinely used biomarker for PC (18–21).

Biomarkers for the prediction of the response to ICIs are highly needed. In this prospective biomarker study, we determined plasma YKL-40 levels in patients with mPC participating in the randomized phase II CheckPAC study (28). The aim of the current study was to investigate whether baseline and on-treatment longitudinal changes in plasma YKL-40 were associated with clinical benefit and survival in patients receiving ICIs combined with stereotactic body radiotherapy (SBRT).

## 2 Methods

### 2.1 Patients

Eighty-four patients with mPC were included in the CheckPAC phase II trial (NCT02866383) at Copenhagen University Hospital – Herlev and Gentofte, Denmark, from November 2016 to December 2019. The patients had experienced disease progression after at least one line of standard chemotherapy and had an Eastern Cooperative Oncology Group performance status (ECOG PS) of 0 or 1. Patients were randomized 1:1 with stratification for ECOG PS to receive SBRT of 15 Gy on day 1 in combination with nivolumab on day 1 and every 2 weeks with or without ipilimumab on day 1 and every 6 weeks. The primary endpoint was clinical benefit rate defined as the proportion of patients with stable disease, partial response, or complete response according to Response Evaluation Criteria in Solid Tumors (RECIST) 1.1. Clinical outcome was evaluated by computed tomography scans every 8 weeks during study treatment and/or at any time when progressive disease was suspected (28). The patients simultaneously participated in the Danish BIOPAC study (NCT03311776) (29). The protocol, amendments, and informed consent form were all approved by the independent ethics committee before study initiation. Signed informed consent was obtained from each participant. Blood samples were collected at baseline, after 2 weeks, and then every 8 weeks until disease progression.

### 2.2 YKL-40 ELISA

In the body, YKL-40 is primarily found in the plasma, hence the level of YKL-40 is referred to as plasma YKL-40. Following peripheral blood collection and centrifugation at 2300 g at 4˚C for 10 minutes within 2 hours of blood sampling, serum was aliquoted and stored at −80˚C until analysis. Serum samples were thawed at room temperature, mixed using a vortex mixer, and centrifuged at 3800 rpm for 10 minutes. YKL-40 was measured using a commercial enzyme-linked immunosorbent assay (ELISA) following the manufacturer’s instructions (MicroVue YKL-40 EIA, catalog number 8020, Quidel, San Diego, CA, USA). The limit of detection for YKL-40 was 20 ng/mL, with an intra-assay coefficient of variation (CV) of ≤ 5% and an inter-assay CV of ≤ 6%. The measurement of YKL-40 was done blinded to patient characteristics and outcomes. Elevated YKL-40 was defined as higher than the 95th age-adjusted percentile of normal (17). The change in plasma YKL-40 on-treatment was defined as follows: decrease: ≥ 40% decrease, stable: < 40% decrease to ≤ 40% increase, and increase: > 40% increase (27). The study report was written according to the REporting recommendations for tumor MARKer prognostic studies (REMARK) guidelines (30).

### 2.3 Statistics

Baseline and longitudinal on-treatment plasma YKL-40 levels were analyzed. A T-test was used for the comparison of two groups after testing for normal distribution. A Mann-Whitney U test was used for the non-parametric comparison of two groups. The Kruskal-Wallis test was used for the comparison of three or more groups. Univariate and multivariate analyses were carried out with Cox proportional hazard models to calculate hazard ratios (HR) for the prediction of OS for elevated baseline YKL-40. Kaplan-Meier curves divided by elevated baseline YKL-40 or change in YKL-40 were used to assess association with progression-free survival (PFS) and OS. A log-rank test was used to compare the survival curves. For correlation analysis, log-transformation was applied for plasma YKL-40, IL-6, IL-8, CA19-9, and C-reactive protein (CRP), and Pearson analysis was performed after testing for normal distribution with the Shapiro-Wilk test. If normal distribution was not observed, Spearman’s correlation coefficient was calculated. A *p*-value < 0.05 was considered significant. All statistical analyses were done using Python software (version 3.8), except for survival analyses analyzed with R software (version 3.6).

## 3 Results

### 3.1 Baseline patient characteristics and plasma YKL-40

A total of 84 patients with mPC were included in the CheckPAC study. Twenty-three patients had a clinical benefit and 61 patients had disease progression as best response to treatment. Eighty-one patients had a baseline blood sample. A more detailed description of the cohort is available in Chen et al. (28). YKL-40 was higher in patients with ECOG PS 1 compared to ECOG PS 0, in patients with a high Modified Glasgow Prognostic Score (mGPS) of 2: CRP > 10 mg/L and albumin < 35 g/L compared to mGPS of 1: CRP > 10 mg/L and albumin ≥ 35 g/L and mGPS of 0: CRP ≤ 10 mg/L and any albumin, and in patients with weight loss ≥ 5% prior to diagnosis (**Table 1**). Additionally, the patients recruited for this study exhibited comorbidities associated with plasma YKL-40 levels as follows: asthma (n = 3), diabetes (n = 2), cardiovascular diseases (n = 2), chronic obstructive pulmonary disease (n = 2), and rheumatic diseases (n = 8).

**Table 1:** Baseline plasma YKL-40 levels in relation to clinical characteristics of the patients included in the CheckPAC study.

Characteristic		Plasma YKL-40 (ng/mL)	
		Median	*P-value*
Sex			
Female, n = 41		219	0.2957
Male, n = 40		220	
ECOG PS			
0, n = 38		180	0.0048
1, n = 43		242	
Weight loss prior to diagnosis			
< 5%, n = 30		188	0.0272
≥ 5%, n = 51		233	
Number of metastatic sites			
1, n = 22		212	0.7597
2, n = 31		239	
3, n = 13		241	
≥ 4, n = 15		199	
mGPS, n (%)			
0, n = 28		177	0.0010
1, n = 38		223	
2, n = 15		472	

Mann-Whitney U test was used to compare medians between two groups, and Kruskal-Wallis test was used for medians between > two groups. Abbreviations: ECOG PS, Eastern Cooperative Oncology Group performance status; mGPS, modified Glasgow Prognostic Score.

### 3.2 Plasma YKL-40 in patients with progressive disease as the best response

**Figure 1:**
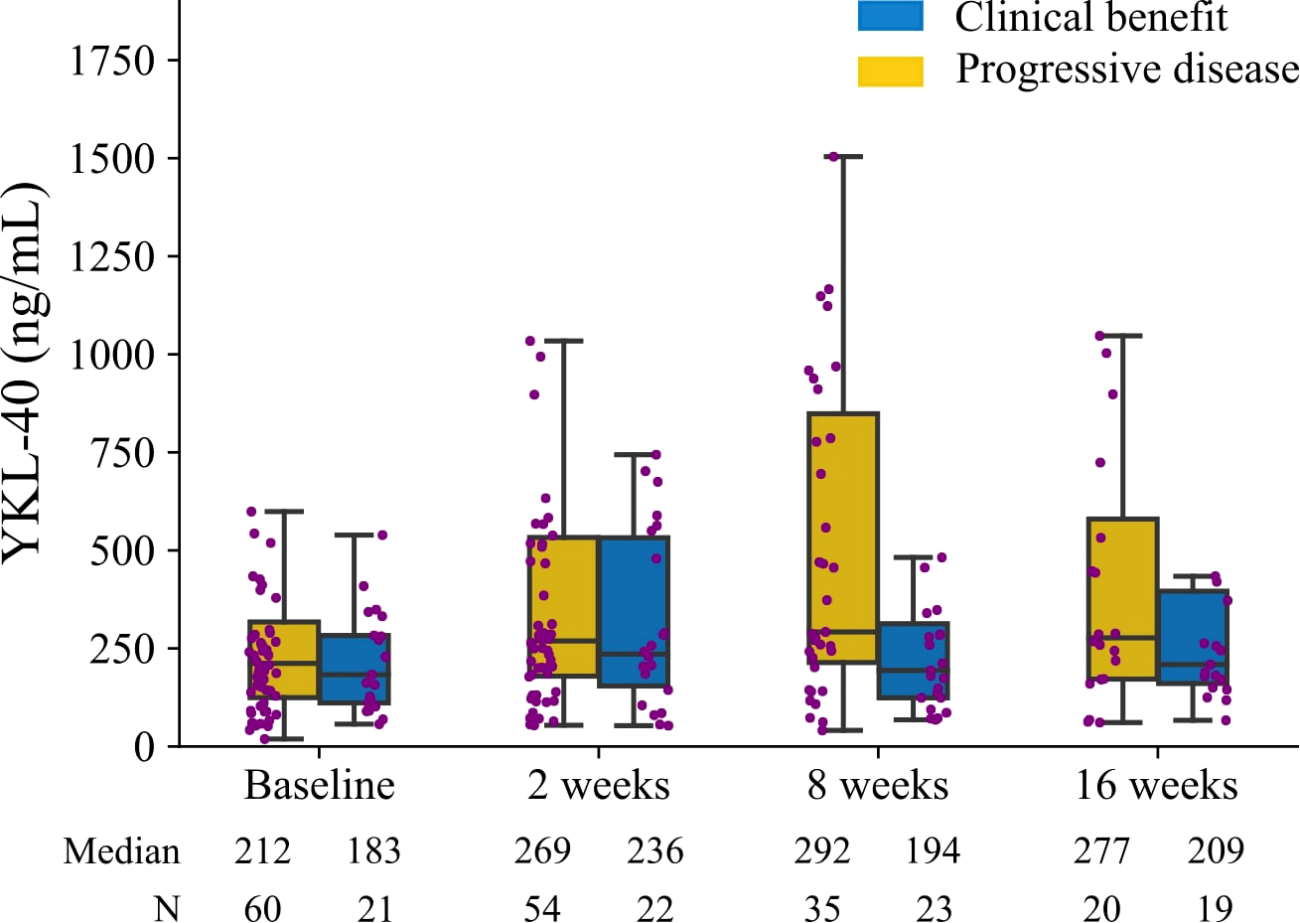
Plasma YKL-40 levels at baseline and after 2, 8, and 16 weeks of treatment. The patients are divided based on response evaluated by computed tomography scans according to RECIST 1.1: clinical benefit defined as stable disease, partial response, or complete response, (blue) and progressive disease (yellow). The boxplot shows the median, upper and lower quartiles, and minimum and maximum. Outliers are not shown. Each dot is an individual patient.

Patients were divided into two groups based on their best response to treatment. There was a tendency toward higher median plasma YKL-40 at baseline, after 2, 8, and 16 weeks in patients with progressive disease than in patients with clinical benefit (**Figure 1**).

### 3.3 Association between plasma YKL-40 and PFS and OS

**Figure 2:**
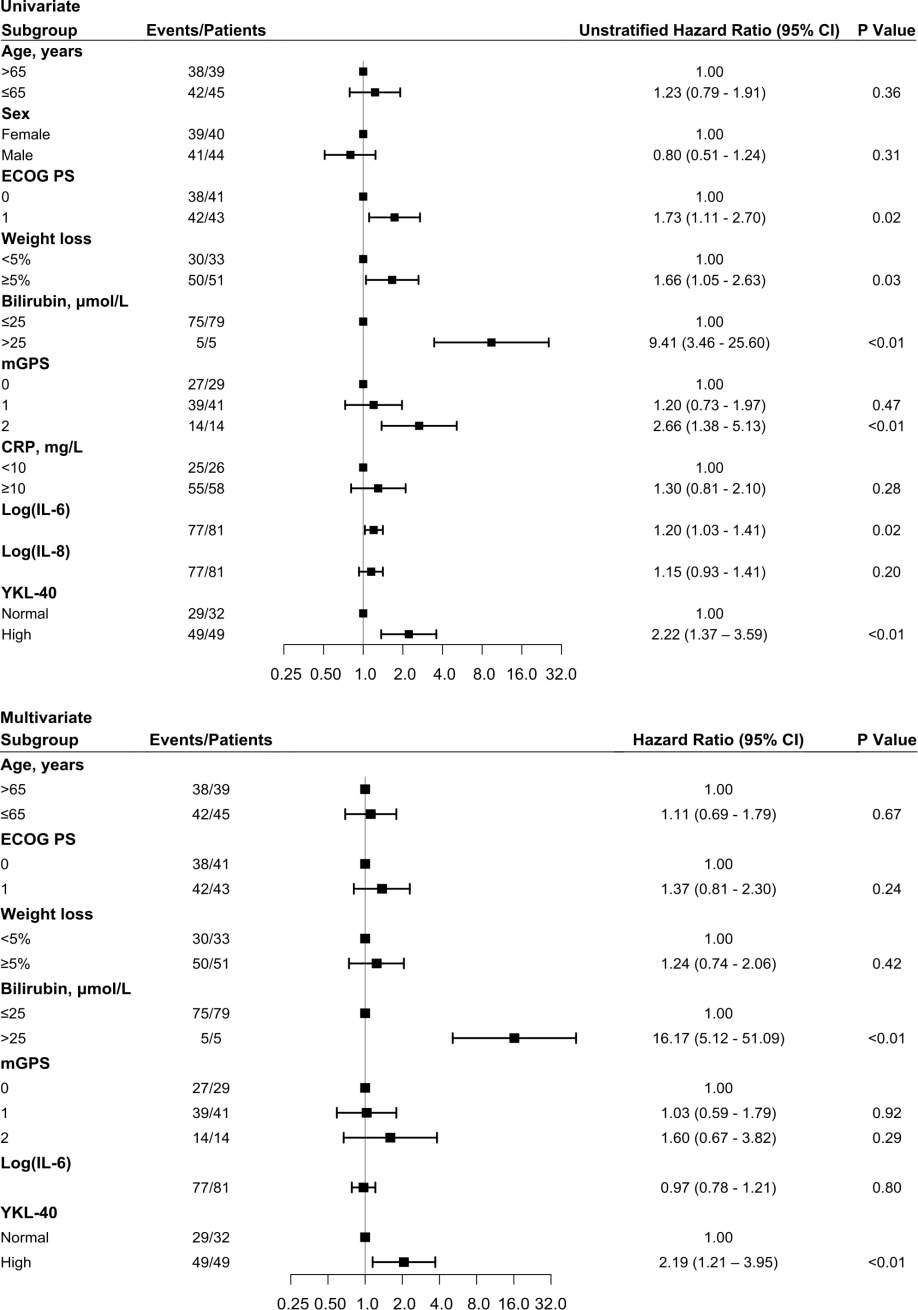
Forest plot with OS HR based on univariate **(top)** and multivariate **(bottom)** Cox regression analysis. Squares represent HR, and bars represent 95% CI. Variables predictive of OS in the univariate Cox regression analysis from Chen et al. (28) were included in this study in addition to baseline YKL-40. CI, confidence intervals; ECOG PS, Eastern Cooperative Oncology Group performance status; mGPS, modified Glasgow Prognostic Score; HR, hazard ratios; OS, overall survival.

Forty-nine patients (60%) had elevated baseline YKL-40. Elevated baseline YKL-40 was prognostic for shorter OS (HR 2.22, 95% confidence interval (CI) 1.37–3.59) (**Figure 2**). After adjusting for age, ECOG PS, weight loss, bilirubin, mGPS, and IL-6, elevated baseline YKL-40 remained a prognostic biomarker for OS (HR 2.19, 95% CI 1.21–3.95) in the Cox proportional hazards regression model (**Figure 2**). A high level of bilirubin (> 25 µmol/L) was also an independent biomarker for OS (HR 16.17, 95% CI 5.12–51.09) (**Figure 2**).

**Figure 3:**
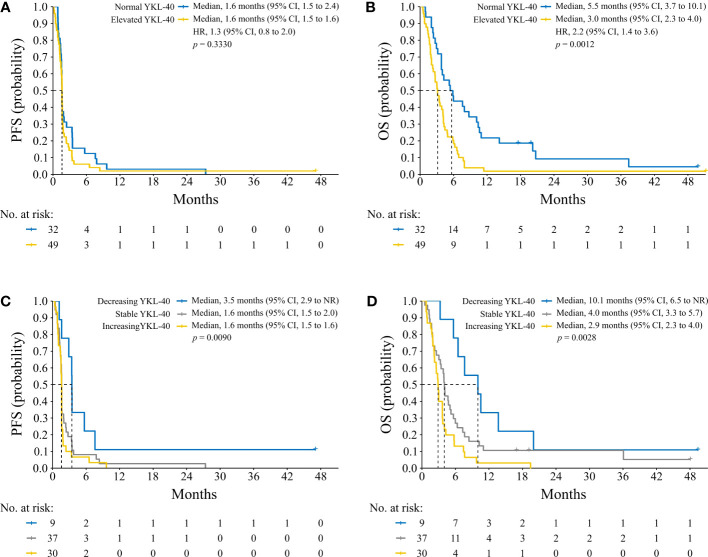
Kaplan Meier plots of PFS **(A, C)** and OS **(B, D)** based on elevated baseline plasma YKL-40 **(A, B)** and change in plasma YKL-40 during treatment **(C, D)**. A log-rank test was used to determine difference between survival curves. CI, confidence interval; NR, not reached; HR, hazard ratio; PFS, progression-free survival; OS, overall survival.

There was no association between elevated baseline plasma YKL-40 and PFS (**Figure 3A**). Patients with elevated baseline plasma YKL-40 had a median OS of 3 months while patients with normal baseline YKL-40 had a median OS of 5.5 months (**Figure 3B**).

### 3.4 Association between changes in plasma YKL-40 during treatment and outcome

Baseline plasma YKL-40 was compared to the on-treatment level after 8 weeks (or 2 weeks if no sample after 8 weeks was available) in the individual patients. Of the nine patients with a decrease in YKL-40, seven (78%) had a clinical benefit (**Supplementary Figure 1**). Patients with a decrease in YKL-40 had longer PFS, a median PFS of 3.5 months (95% CI 2.9 to not reached (NR)) versus 1.6 months (95% CI 1.5–2.0) for stable levels and 1.6 months (95% CI 1.5–1.6) for increasing levels (**Figure 3C**). Patients with a decrease in YKL-40 also had longer OS, a median OS of 10.1 months (95% CI 6.5 to NR) versus 4 months (95% CI 3.3–5.7) for stable levels and 2.9 months (95% CI 2.3–4.0) for increasing levels (**Figure 3D**).

**Figure 4:**
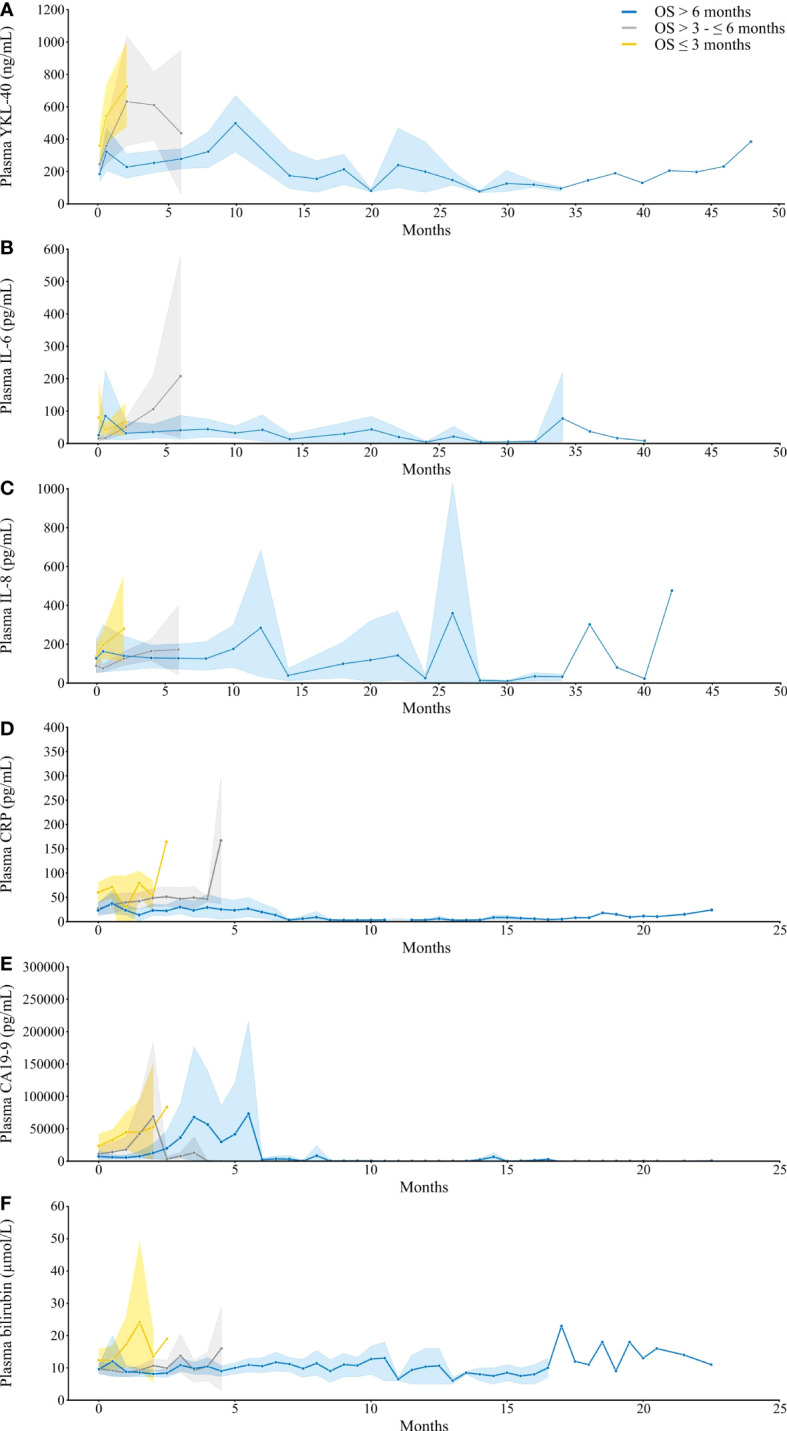
Plots illustrating plasma YKL-40 **(A)**, IL-6 **(B)**, IL-8 **(C)**, CRP **(D)**, CA19-9 **(E)**, and bilirubin **(F)** over time from baseline to last sample. The lines represent the mean values and the shaded area the 95% CI. If there is no shaded area, only one value was available. The patients are divided into three groups based on OS: ≤ 3 months (yellow, n = 30 **(A, B, C)** or n = 32 **(D–F)**), > 3 to ≤ 6 months (grey, n = 25 **(A, B, C, E)** or n = 26 **(D, F)**), and > 6 months (blue, n = 21 **(A, C)**, n = 22 **(B)** or n = 26 **(D–F)**). IL-6, interleukin 6; IL-8, interleukin 8; CRP, C-reactive protein; CA19-9, carbohydrate antigen 19-9; CI, confidence interval; OS, overall survival.

Plasma CRP, CA19-9, and bilirubin were routinely measured in the patients, and IL-6 and IL-8 were analyzed as part of the BIOPAC study (**Supplementary Methods**). Plasma YKL-40, IL-8, IL-9, CRP, CA-19, and bilirubin levels during treatment were investigated in patients divided into OS ≤ 3 months, > 3 to ≤ 6 months, and > 6 months survival (**Figure 4**), but due to the large variation in the groups, no statistical model was applied. Individual levels of these five biomarkers are shown in **Supplementary Figure 2**. Two patients had an exceptionally long survival. In these two patients, increased plasma YKL-40 levels were occasionally found at specific timepoints related to other conditions, including arthritis symptoms and a liver abscess. But, in general, stable levels of plasma YKL-40 were observed (**Supplementary Figure 3**).

### 3.5 Correlations between plasma YKL-40 and CA19-9, CRP, bilirubin, IL-6, IL-8, and tissue PD-L1 expression

**Figure 5:**
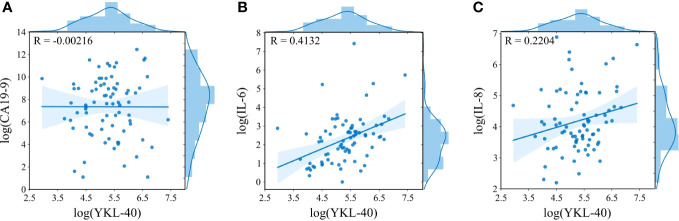
Relationships between baseline plasma YKL-40 and CA19-9 **(A)**, IL-6 **(B)**, and IL-8 **(C)**. On the x- and y-axis, histograms show the distribution of the biomarkers after log-transformation. A Pearson correlation coefficient was calculated (upper left corner). CA19-9, carbohydrate antigen 19-9; IL-6, interleukin 6; IL-8, interleukin 8.

**Figure 6:**
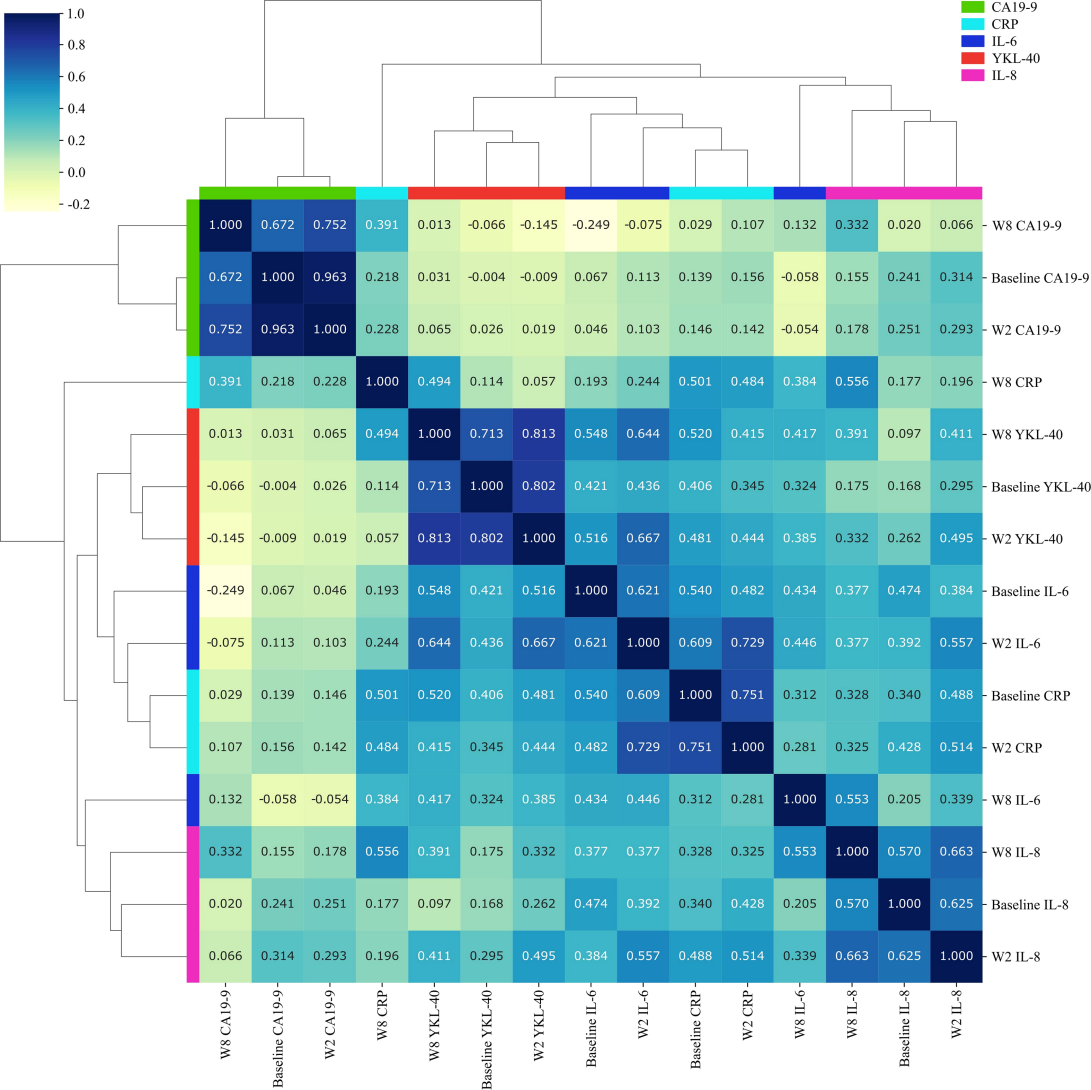
Heatmap illustrating relationships between plasma YKL-40, IL-6, IL-8, CRP, and CA19-9 at baseline and after 2 and 8 weeks of treatment. Spearman’s correlations were calculated (scale: upper left corner). The clustering of biomarkers was based on Spearman’s correlation analysis. IL-6, interleukin 6; IL-8, interleukin 8; CRP, C-reactive protein; CA19-9, carbohydrate antigen 19-9; W2, week 2; W8, week 8.

There was no correlation between baseline YKL-40 and CA19-9 and IL-8 (**Figures 5A, C**), while there was a trend toward a positive correlation between YKL-40 and IL-6 (**Figure 5B**). Spearman’s correlation coefficients between all biomarkers were calculated for samples collected at baseline and after 2 weeks and 8 weeks. As can be seen in **Figure 6**, two overall different clusters were observed: CA 19-9 cluster (green) and an inflammatory biomarker cluster (remaining colors). For the inflammation biomarkers, YKL-40 (red) and IL-8 (purple) clustered alone compared to CRP and IL-6 (blue colors), for which there was no tendency for clustering within biomarker type nor timepoint. In conclusion, there was a trend toward a positive association between the different inflammation biomarkers that was strongest between IL-6 and CRP after 2 weeks of treatment (ρ = 0.73).

No correlation was found between baseline plasma bilirubin and YKL-40 (ρ = 0.14) nor tissue PD-L1 expression and baseline YKL-40 (ρ = −0.25) (data not shown).

## 4 Discussion

In this prospective biomarker study including patients with mPC treated with a combination of ICIs and SBRT, we found that elevated baseline plasma YKL-40 was an independent predictor of short OS and that a decrease in plasma YKL-40 during treatment was associated with longer PFS and OS. To our knowledge, this is the first study of plasma YKL-40 in cancer patients treated with ICIs and radiotherapy.

Current biomarkers for prediction of clinical benefit from checkpoint inhibitors include high tumor PD-L1 expression, high tumor mutational burden (TMB), and an inflamed T cell signature (31). PC is characterized by low PD-L1 expression, low TMB, and low T cell infiltration, indicating a low probability of clinical benefit from ICIs (32). Recently, we have demonstrated that a combination treatment strategy using nivolumab, ipilimumab, and a single dose of SBRT can deliver clinical benefit in patients with chemotherapy-resistant mPC (28).

In the present study, we found no association between baseline plasma YKL-40 and treatment response to ICIs. Prior to inclusion in the CheckPAC study, patients had progressed after being treated with one to three lines of chemotherapy. It is therefore unknown whether an association exists between baseline plasma YKL-40 and clinical benefit in treatment-naive patients receiving ICIs, highlighting the importance of investigating plasma YKL-40 before treatment start.

There is no well-defined cut-off for clinically relevant changes in inflammatory biomarker levels in patients treated with immunotherapy, but a 40% cut-off has been suggested (28, 33). We found that a ≥ 40% decrease in plasma YKL-40 during treatment was associated with longer PFS and OS. Patients that experienced a clinical benefit were largely represented in the groups that either had a ≥ 40% decrease or stable plasma YKL-40, suggesting that changes in plasma YKL-40 could be a predictive marker for clinical benefit from ICIs combined with radiotherapy. A recent, small study of 11 patients with lung cancer treated with ICI similarly showed that partial response to treatment correlated with a decrease in plasma YKL-40 during treatment (34). Others have suggested that YKL-40 mediates immune suppression, thereby reducing the efficacy of ICIs through inhibition of the cytotoxicity of T and NK lymphocytes (8, 9, 11). A decrease in YKL-40 could indicate a favorable clinical outcome after ICI treatment; however, the underlying mechanism remains unknown. Studies including a larger number of treatment-naïve patients with more responsive cancer types could be valuable to define an appropriate cut-off and investigate the predictive value of YKL-40 for response to immunotherapy.

Plasma YKL-40 was not correlated with CA19-9, a clinical tumor burden marker in PC (35). Whether the decrease in YKL-40 in patients with clinical benefit is due to lower tumor burden, i.e. killing of YKL-40 producing cells, is unknown. However, only a minority of patients with clinical benefit had a reduction of tumor mass (28). A high level of bilirubin was also an independent biomarker for OS. Elevated plasma bilirubin levels have been associated with poorer survival in patients with PC (36), and treatment with immunotherapy and SBRT has been shown to result in elevated levels of bilirubin (37) suggesting future studies of bilirubin as a potential biomarker. There was no correlation between plasma bilirubin and YKL-40. Positive correlations between YKL-40 and other biomarkers involved in cancer-promotion or systemic inflammation (IL-6, IL-8, and CRP) were identified in this study. Secretion of IL-6 by immune cells has been shown to promote YKL-40 transcription through activation of a range of transcription factors including NF-κB and STAT3 (1, 38). Moreover, YKL-40 stimulates IL-8 secretion in human colorectal cancer cells (39), and YKL-40 secreted by cancer-associated fibroblasts stimulates IL-8 production through binding to the IL-13 receptor α2 (40). Inflammation and cytokine profiles combining multiple biomarkers have been investigated in patients treated with ICIs and were found to be predictive of response and survival (41). An ongoing study from our group will evaluate if a combination of 92 plasma proteins will provide better predictive and prognostic information about the patients in the CheckPAC study.

YKL-40 has been suggested to increase the expression of immune checkpoints (9, 42), and recently, a study found a positive correlation between the expression of YKL-40 in immune cells and PD-L1 expression in tissue sections from patients with colorectal cancer (43). However, in our study, we found no correlation between plasma YKL-40 and tissue PD-L1 expression at baseline.

We only investigated patients with PC, which is known for its resistance to ICIs. Therefore, our findings cannot necessarily be extrapolated to patients with other cancer types, including cancers with more favorable biology and responsiveness to immunotherapy. All patients received radiotherapy combined with ICIs. Thus, the prognostic value of YKL-40 level in patients treated with immunotherapy alone is still unknown. Additionally, the patient population was quite small and heterogeneous regarding tumor burden and numbers of prior treatments, limiting generalizability. Furthermore, YKL-40 is not a cancer-specific biomarker, and it is also elevated in non-malignant diseases characterized by inflammation such as infections, autoimmune- and fibrotic diseases (2). The limited number of patients within each disease group restricts our ability to thoroughly investigate the role of the comorbidities. However, considering the advanced stage of PC in the patients included in this study, the impact of the comorbidities on plasma YKL-40 is likely less compared to the influence of PC. Additionally, ICIs can cause a variety of inflammatory side effects related to the disruption of immunologic homeostasis and T cell tolerance (44), potentially causing increased levels of plasma YKL-40.

In conclusion, this study contributes new knowledge regarding plasma YKL-40 as a biomarker for clinical benefit in a cohort of patients with mPC receiving ICIs combined with radiotherapy. These results warrant further investigation in larger, multicenter studies across different cancer types more responsive to ICIs.

## Data availability statement

The raw data supporting the conclusions of this article will be made available by the authors, without undue reservation.

## Ethics statement

The studies involving humans were approved by The Danish Ethics Committee and the Danish Data Protection Agency. The studies were conducted in accordance with the local legislation and institutional requirements. The participants provided their written informed consent to participate in this study.

## Author contributions

Conception and methodology: AJ, DM, DN, IC. Collection and assembly of data: AJ, SN, ST, IC. Statistical analysis and interpretation: AJ, SN, JH, MB, SB, DM, DN, IC. Manuscript writing: AJ, IC. All authors contributed to the article and approved the submitted version.
