# Supplementary material for: Plasma YKL-40 is associated with prognosis in patients with metastatic pancreatic cancer receiving immune checkpoint inhibitors in combination with radiotherapy
Source: Front Immunol. 2023 Sep 7;14:1228907. doi: 10.3389/fimmu.2023.1228907 (PMC10513102; doi:10.3389/fimmu.2023.1228907)
Supplement: Supplementary file 1 [file DataSheet_1.docx]

Supplementary Material

Plasma YKL-40 is associated with prognosis in patients with metastatic pancreatic cancer receiving immune checkpoint inhibitors in combination with radiotherapy

Astrid Z. Johansen^1^, Sif I. Novitski^2^, Jessica X. Hjaltelin^2^, Susann Theile^3^, Mogens K. Boisen^3^, Søren Brunak^2^, Daniel H. Madsen^1,4^, Dorte L. Nielsen^3,5^, Inna M. Chen^3*^

*** Correspondence:** Inna M. Chen: inna.chen@regionh.dk

# Supplementary Methods

**1.1 Measurements of plasma CA19-9, CRP, IL-6, and IL-8**

Concentrations of CA19-9 and CRP were determined as part of the routine blood analyses. CA19-9 was measured with a solid-phase, two-site sequential chemiluminescent immunometric assay (Immulite 2000 GI-MA assay, catalog number L2KG12, Siemens). Imprecision was monitored with two internal controls at 16 and 83 kU/L with CVs of 8% and 9%. Accuracy was monitored within the standard UK NEQAS program.

CRP was measured using a CRP Ultra ready-to-use, liquid assay reagent by an immunoturbidimetric method on a fully automated chemistry analyzer (Kit-test SENTINEL CRP Ultra (UD), 11508 UD-2.0/02 2015/09/23) according to the manufacturer's instructions. The detection limit is 0.3 mg/L, with an intra-assay CV of 3% and an inter-assay CV of < 15%.

Plasma IL-6 concentration was measured in duplicates with high-sensitive ELISA according to the manufacturer's instructions (high-sensitive IL-6 ELISA, catalog number HS600, R&D Systems®, Abingdon, United Kingdom). The detection limit is 0.1 ng/L, with an intra-assay CV of ≤ 7.8% and an inter-assay CV of ≤ 11.2%.

Plasma IL-8 concentration was measured in duplicates with ELISA according to the manufacturer's instructions (IL-8 ELISA, catalog number D8000C, R&D Systems®, Abingdon, United Kingdom). The detection limit is 7.5 ng/L, with an intra-assay CV of ≤ 6.5% and an inter-assay CV of ≤ 8.1%.

**1.2 Quantification of PD-L1 tissue expression**

Biopsy samples obtained at baseline were formalin-fixed and paraffin-embedded. Sections with more than 100 tumor cells based on a hematoxylin and eosin stain underwent immunohistochemical (IHC) staining for programmed death-ligand 1 (PD-L1) expression (PD-L1 IHC 28-8 pharmDx, catalog number SK005, Dako). Two pathologists calculated independently a combined positive score for PD-L1 defined as the number of positive cells (both tumor and immune cells) divided by the total count of tumor cells multiplied by 100.

Supplementary material is not typeset so please ensure that all information is clearly presented, the appropriate caption is included in the file and not in the manuscript, and that the style conforms to the rest of the article.

# Supplementary Figures and Tables

## Supplementary Figures

**
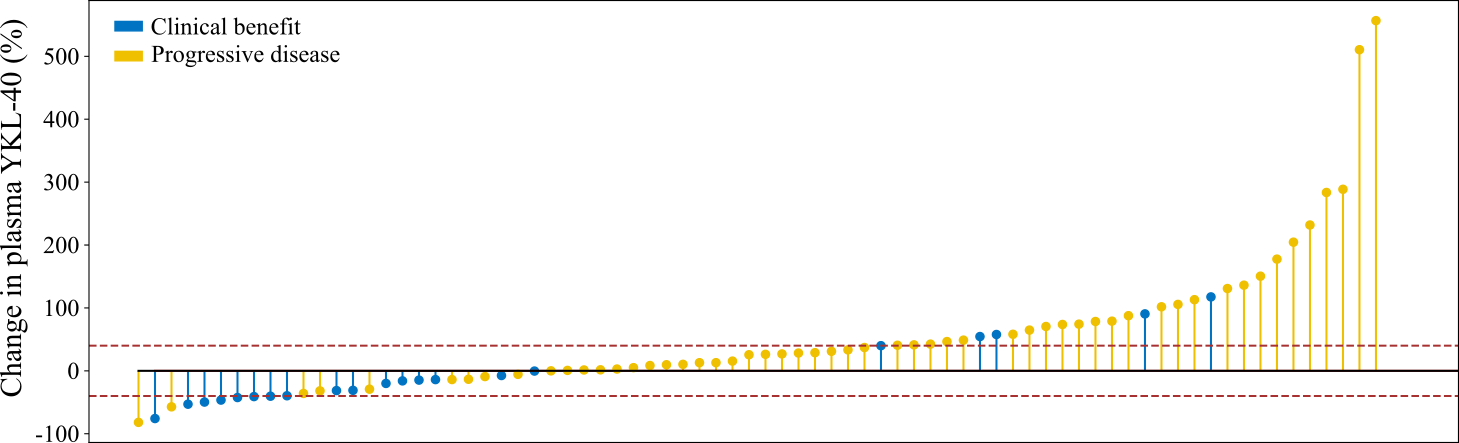
**

**Supplementary Figure 1.** Plot illustrating the change in plasma YKL-40 from baseline to 8 weeks (or 2 weeks if no sample after 8 weeks was available, n = 18) in the individual patients. The patients are ranked from largest decrease to largest increase. The patients are colored based on response: clinical benefit (blue) and progressive disease (yellow). The dashed lines indicate a 40% decrease or increase.

**
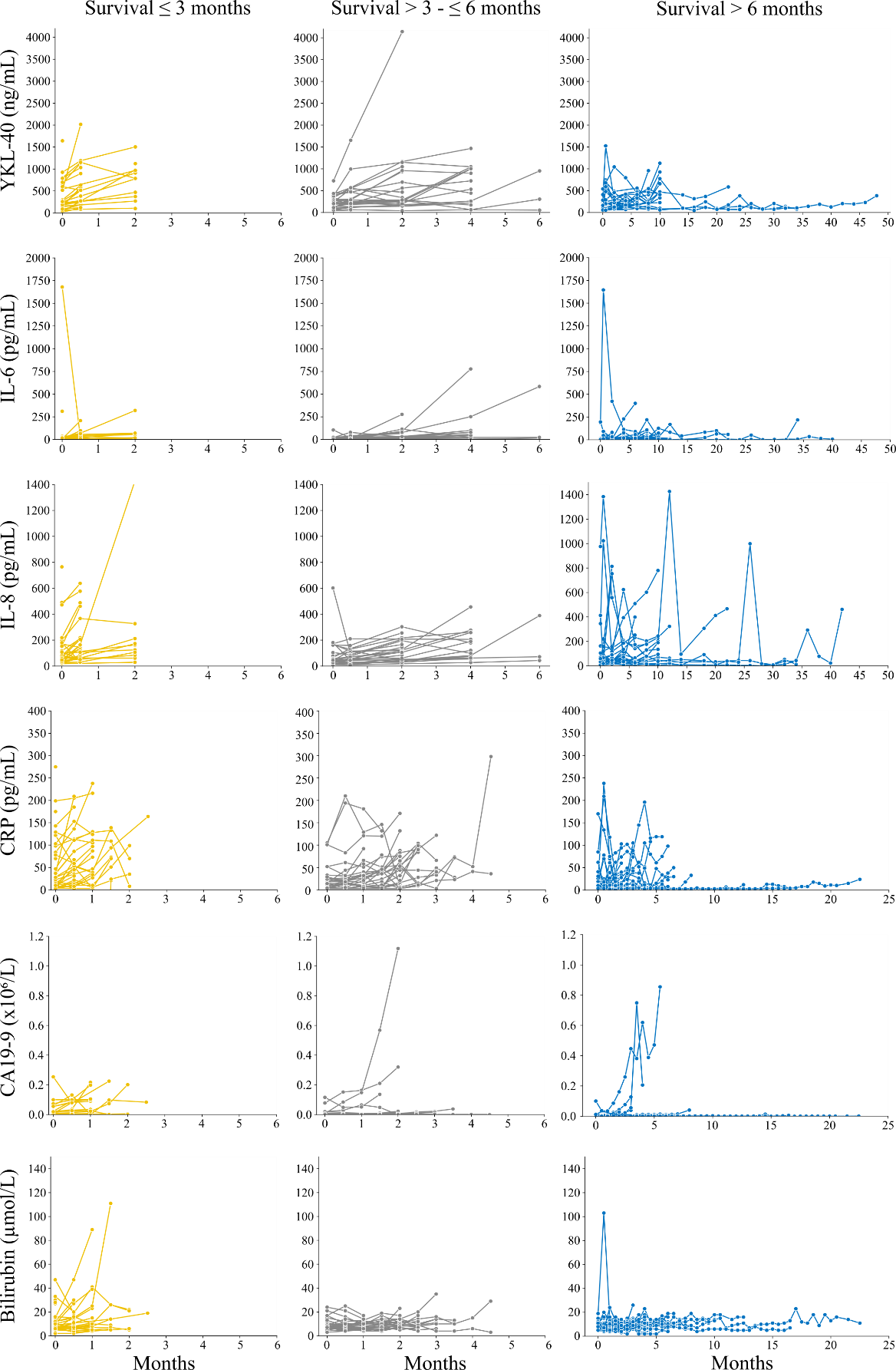
**

**Supplementary Figure 2.** Plots illustrating the plasma levels of YKL-40, IL-6, IL-8, CRP, CA19-9 and bilirubin in individual patients over time. The patients are divided into three groups based on OS: ≤ 3 months (yellow), > 3 to ≤ 6 months (grey), and > 6 months (blue). Abbreviations: IL-6, interleukin 6; IL-8, interleukin 8; CRP, C-reactive protein; CA19-9, cancer-antigen 19-9.

**
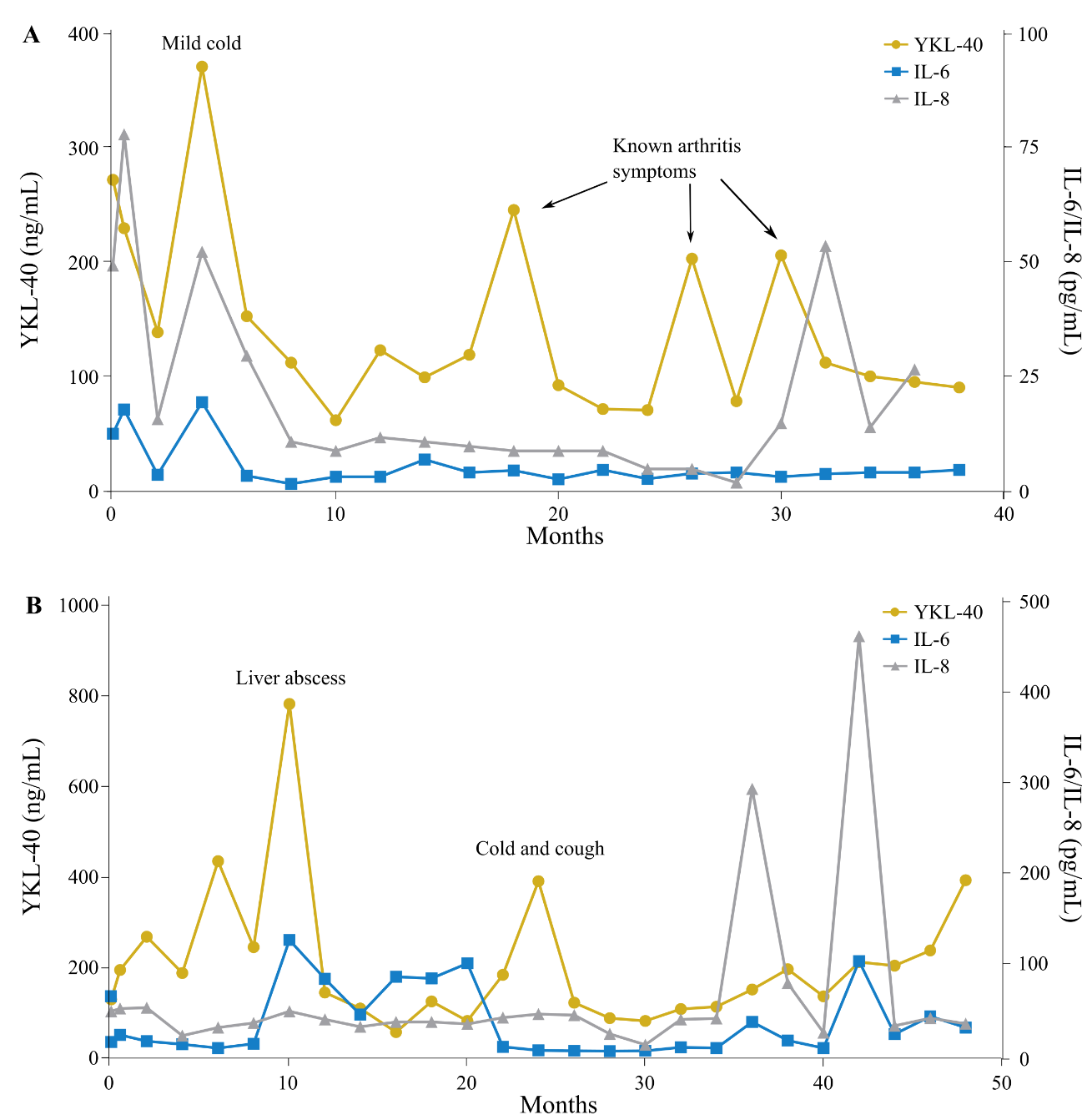
**

**Supplementary Figure 3.** Plasma YKL-40, IL-6, and IL-8 over time in exceptional responder 1 **(A)** and 2 **(B)**. Abbreviations: IL-6, interleukin 6; IL-8, interleukin 8.

**Supplementary Table 1**. The REMARK Checklist.

| **Item to be reported** | | **Page no.** |
| --- | --- | --- |
| **INTRODUCTION** | |  |
| 1 | State the marker examined, the study objectives, and any pre-specified hypotheses. | 2 |
| **MATERIALS AND METHODS** | |  |
| *Patients* | |  |
| 2 | Describe the characteristics (e.g., disease stage or co-morbidities) of the study patients, including their source and inclusion and exclusion criteria. | 2-3, Table 1 |
| 3 | Describe treatments received and how chosen (e.g., randomized or rule-based). | 2 |
| *Specimen characteristics* | |  |
| 4 | Describe type of biological material used (including control samples) and methods of preservation and storage. | 2-3 |
| *Assay methods* | |  |
| 5 | Specify the assay method used and provide (or reference) a detailed protocol, including specific reagents or kits used, quality control procedures, reproducibility assessments, quantitation methods, and scoring and reporting protocols. Specify whether and how assays were performed blinded to the study endpoint. | 2-3 |
| *Study design* | |  |
| 6 | State the method of case selection, including whether prospective or retrospective and whether stratification or matching (e.g., by stage of disease or age) was used. Specify the time period from which cases were taken, the end of the follow-up period, and the median follow-up time. | 2-3 |
| 7 | Precisely define all clinical endpoints examined. | NA |
| 8 | List all candidate variables initially examined or considered for inclusion in models. | Table 1, Figure 2 |
| 9 | Give rationale for sample size; if the study was designed to detect a specified effect size, give the target power and effect size. | NA |
| *Statistical analysis methods* | |  |
| 10 | Specify all statistical methods, including details of any variable selection procedures and other model-building issues, how model assumptions were verified, and how missing data were handled. | 3 |
| 11 | Clarify how marker values were handled in the analyses; if relevant, describe methods used for cut-point determination. | 3 |
| **RESULTS** | |  |
| *Data* | |  |
| 12 | Describe the flow of patients through the study, including the number of patients included in each stage of the analysis (a diagram may be helpful) and reasons for dropout. Specifically, both overall and for each subgroup extensively examined report the numbers of patients and the number of events. | NA |
| 13 | Report distributions of basic demographic characteristics (at least age and sex), standard (disease-specific) prognostic variables, and tumor marker, including numbers of missing values. | Table 1 |
| *Analysis and presentation* | |  |
| 14 | Show the relation of the marker to standard prognostic variables. | Figures 2, 5, 6 |
| 15 | Present univariable analyses showing the relation between the marker and outcome, with the estimated effect (e.g., hazard ratio and survival probability). Preferably provide similar analyses for all other variables being analyzed. For the effect of a tumor marker on a time-to-event outcome, a Kaplan-Meier plot is recommended. | Figures 2, 3 |
| 16 | For key multivariable analyses, report estimated effects (e.g., hazard ratio) with confidence intervals for the marker and, at least for the final model, all other variables in the model. | Figure 2 |
| 17 | Among reported results, provide estimated effects with confidence intervals from an analysis in which the marker and standard prognostic variables are included, regardless of their statistical significance. | NA |
| 18 | If done, report results of further investigations, such as checking assumptions, sensitivity analyses, and internal validation. | NA |
| **DISCUSSION** | |  |
| 19 | Interpret the results in the context of the pre-specified hypotheses and other relevant studies; include a discussion of limitations of the study. | 5-8 |
| 20 | Discuss implications for future research and clinical value. | 8 |
